# Familial Patterns in Acute Lymphoblastic Leukemia: Lessons From Three Siblings

**DOI:** 10.1002/ccr3.71125

**Published:** 2025-10-05

**Authors:** Alireza Khanahmad, Mohammad Hossein Khazaee‐Nasirabadi, Naeem Nikpour, Mahmood Khosravi

**Affiliations:** ^1^ Student Research Committee, Afzalipour Faculty of Medicine Kerman University of Medical Sciences Kerman Iran; ^2^ Department of Hematology and Medical Laboratory Sciences, Faculty of Allied Medicine Kerman University of Medical Sciences Kerman Iran; ^3^ Department of Internal Medicine, Hematology and Medical Oncology Division Kerman University of Medical Sciences Kerman Iran; ^4^ Department of Medical Laboratory Sciences, School of Allied Medical Sciences Arak University of Medical Sciences Arak Iran

**Keywords:** acute leukemia, B‐lymphoblastic leukemia/lymphoma, case report, De‐novo, familial cancer

## Abstract

Acute lymphoblastic leukemia is a sporadic condition and has been reported to be familial in less than 5% of cases. Here, we present the clinical, genetic, treatment, and pathologic features of three siblings with precursor B acute lymphoblastic leukemia by the order of occurrence, suggesting a potential involvement of germline mutations in the pathogenesis of the disease. Their parents denied consanguinity as well as any familial history of syndromic diseases or neoplasms. Cases 1 and 2 have experienced a relapse after achieving remission, and case 3 has been diagnosed in recent years. Recurrent cytogenetic abnormalities shared across Cases 2 and 3 raised the hypothesis that monosomy 20 may be a susceptible marker in familial B‐ALL. HLAs were fully matched for Cases 2 and 3; so, Case 3 donated stem cells to his older brother (Case 2) 3 months before his own involvement. Today, these cases are in complete remission of the disease. This study supports genetic counseling and targeted germline testing in familial ALL cases and argues for cautious consideration of related donors in such cases.

**Summary**

- This report describes an extremely rare and tragic familial occurrence of acute lymphoblastic leukemia.
- This report discusses the underlying genetic abnormalities and highlights that high‐throughput genetic testing and genetic counseling are warranted when selecting a related donor for stem cell transplantation in such settings.

## 1 Introduction

Acute lymphoblastic leukemia (ALL) is the most common childhood cancer, making up about one‐third of all pediatric malignancies. ALL arises from clonal proliferation of immature lymphoid progenitor cells [1, 2]. The peak incidence of ALL is reported between ages 2 and 5 years, and the annual incidence rate is 36.2 per 1 million individuals [3]. Most cases of ALL have no known cause, but an increase in incidence has been associated with certain environmental or genetic risk factors [4]. While traditionally, ALL is reported as a sporadic disease caused by acquired somatic mutations [5], emerging evidence highlights the significant role of inheritable genetic risk factors in its development. Approximately 20% of familial ALL cases stem from inherited syndromic defects and germline polymorphisms. Besides its high prevalence in early childhood, congenital occurrence suggests that inheritable risk factors are involved [6, 7]. Inherited syndromic defects affecting DNA repair (e.g., Bloom), tumor suppression (e.g., Neurofibromatosis, Li‐Fraumeni, and Down syndromes), apoptosis (e.g., autoimmune lymphoproliferative syndrome), immune system function (e.g., severe combined immunodeficiency), and syndromic defects that lead to second mutations (e.g., familial platelet disorder) may cause familial cases of leukemias [8, 9]. Germline mutations (PAX5, ETV6, TP53, IKZF1) and polymorphisms (ARID5B, CDKN2A/B) are less common but, together with the mentioned syndromes, account for approximately one‐fifth of all familial ALL cases [6].

Here, we adhere to the CARE guidelines to describe the demographic, clinical, genetic, treatment, and pathologic features of three Iranian siblings who developed precursor B‐lymphoblastic leukemia/lymphoma in the order of occurrence.

## 2 Case History/Examination

To uncover the genetic and environmental predisposing factors, the past medical history of the parents, affected, and non‐affected siblings was explored. It was demonstrated that besides three ALL‐affected siblings, one other sibling died of kernicterus at the age of 7, and the other child suffers from schizophrenia. The mother of the affected siblings reported no significant viral infections during her pregnancies. There was no history of ionizing radiation exposure or chemotherapy use prior to or during pregnancy, and she did not use any chronic medications aside from routine prenatal vitamins. The family resides in a rural area with no known exposure to industrial carcinogens or agricultural pesticides, and there was no reported household smoking or occupational toxin exposure. According to pediatric records, none of the three siblings experienced severe viral illnesses during infancy or early childhood. The lack of significant environmental or infectious exposures in this family supports the likelihood of a primary genetic predisposition. All family members have a completely normal diet. Consanguinity, history of syndromic defects, or neoplasms among first‐ and second‐degree relatives were denied.

## 3 Differential Diagnosis, Investigations, and Treatment

### 3.1 Case 1

A 10‐year‐old female was admitted to the pediatric hematology oncology ward of Afzalipour Hospital in Kerman, Iran, due to a suspicion of acute leukemia. She had a history of anorexia, weight loss, abdominal pain, fever, and nosebleeds. Laboratory studies showed anemia, thrombocytopenia, and leukopenia, along with mild splenomegaly, which indicated the need for bone marrow aspiration and biopsy. It should be noted that the initial diagnosis occurred several years ago, at a time when cytogenetic analyses were not routinely available, and their diagnostic/prognostic value in ALL was not fully established. The girl was diagnosed with precursor B‐ALL and achieved hematological remission after receiving the AIEOP‐BFM ALL protocol. Eight years after her initial diagnosis, the patient was re‐admitted with pancytopenia, characterized by hemoglobin (Hb) levels of 8.7 g/dL, a white blood cell (WBC) count of 1.3 × 10^3^/μL, and a platelet (Plt) count of 14 × 10^3^/μL. Clinical symptoms included hip pain, inability to walk, and neutropenia. Hypoplastic areas were observed at the gray‐white matter junction, which may indicate underlying brain involvement. The patient then underwent a whole‐body scan 3 h after an IV injection of ^20m^Ci ^99m^TC‐MDP. The results showed increased radiotracer uptake with an irregular pattern in the femoral shafts throughout the pelvis, including the sacroiliac joints (SIJ) and the upper thoracic spine. Additionally, bilateral relative hyperactivity in the proximal and distal parts of some long tubular bones was reported. These findings were suggestive of either marrow reaction or an infiltrative process. A focal zone of mild tracer uptake in the right frontal region was likely due to an intracranial lesion. Bone marrow aspiration and biopsy showed relapse, and an intrathecal (IT) chemotherapy treatment protocol, which included methotrexate, hydrocortisone, and cytarabine, was administered to the patient.

### 3.2 Case 2

**FIGURE 1:**
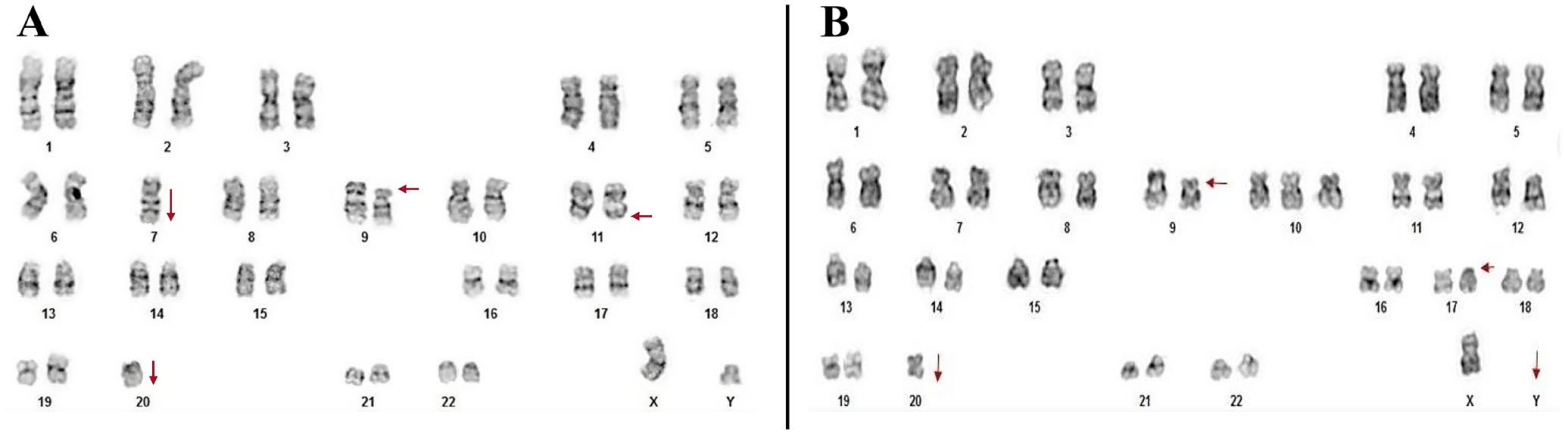
(A) Complex karyotype in Case 2: −7, del (9)(p22), del (11)(q23), −20; (B) Complex karyotype in Case 3: del (9)(p22), +10, del (17)(p3), −20, −Y.

Three years after the initial diagnosis of case 1, her older brother was admitted to the hematology oncology ward of Afzalipour Hospital in Kerman, Iran, with epistaxis as the chief complaint. He was a 17‐year‐old male with anemia, thrombocytopenia, and leukocytosis, along with mild splenomegaly, which indicated further bone marrow investigation. Immunophenotyping showed the expression of TdT, CD20, CD10, and CD22. The expression of CD34 and CD4 was reported at 51% and 16%, respectively. There was no expression of cytoplasmic CD3 (pan T‐cell marker). Overall, acute precursor B‐lymphoblastic leukemia was diagnosed. He was started on hyper‐CVAD treatment protocol and achieved complete remission (CR). Ten years after his initial diagnosis, he returned with epistaxis and myalgia. CBC and differential WBC count showed a mild leukopenia (3200/μL) with 50% lymphocytes. Platelet count was also decreased (64,000/μL), and the presence of giant platelets was reported. Bone marrow evaluation illustrated sheets of blastic cells positive for CD10, CD20, TdT, HLA‐DR, and co‐expression of HLA‐DR & TdT, indicating relapsed‐ALL. Qualitative assessment using the Real‐time PCR technique showed a negative fusion transcript p210 of BCR‐ABL/t(9;22)(q34;q11). However, the complex abnormal karyotype with several poor prognostic criteria, including hypodiploidy, monosomy 7, KMT2a gene rearrangement, and monosomy 20, made therapeutic reinforcement and bone marrow transplantation inevitable (Figure 1A). HLA typing showed a full match of HLA with his younger brother (Table 1). To achieve CR before transplantation, he started on Endoxan (550 mg), Vincristin (2 mg), Doxorubicin (90 mg), and Rituximab (700 mg). After 40 days, he continued with R‐MTX‐CYTOSAR. Finally, a successful bone marrow transplant was done.

**TABLE 1:** Results of low‐resolution HLA‐typing showed a full match for Case 2 and his younger brother, who developed ALL after transplantation and was labeled as Case 3.

Label	HLA‐I			HLA‐II		
	A	B	C	DRB1	DRB3,4,5	DQB1
Father	02, − Bw6	45, 35	—	—	—	—
Mother	24, 30 Cw7/Bw6	14, 18	—	—	—	—
Older Brother	02, 30	14, 45	—	03, 15	P, N, P	—
Case 1	02, − Bw6	14, −	—	—	—	—
Case 2	02, 24 Cw7/Bw6	18, 45	07, 16	01, 03	DRB3	04, 05
Case 3	02, 24 Cw7, Bw6	18, 45	07, 16	01, 03	DRB3	04, 05

### 3.3 Case 3

**FIGURE 2:**
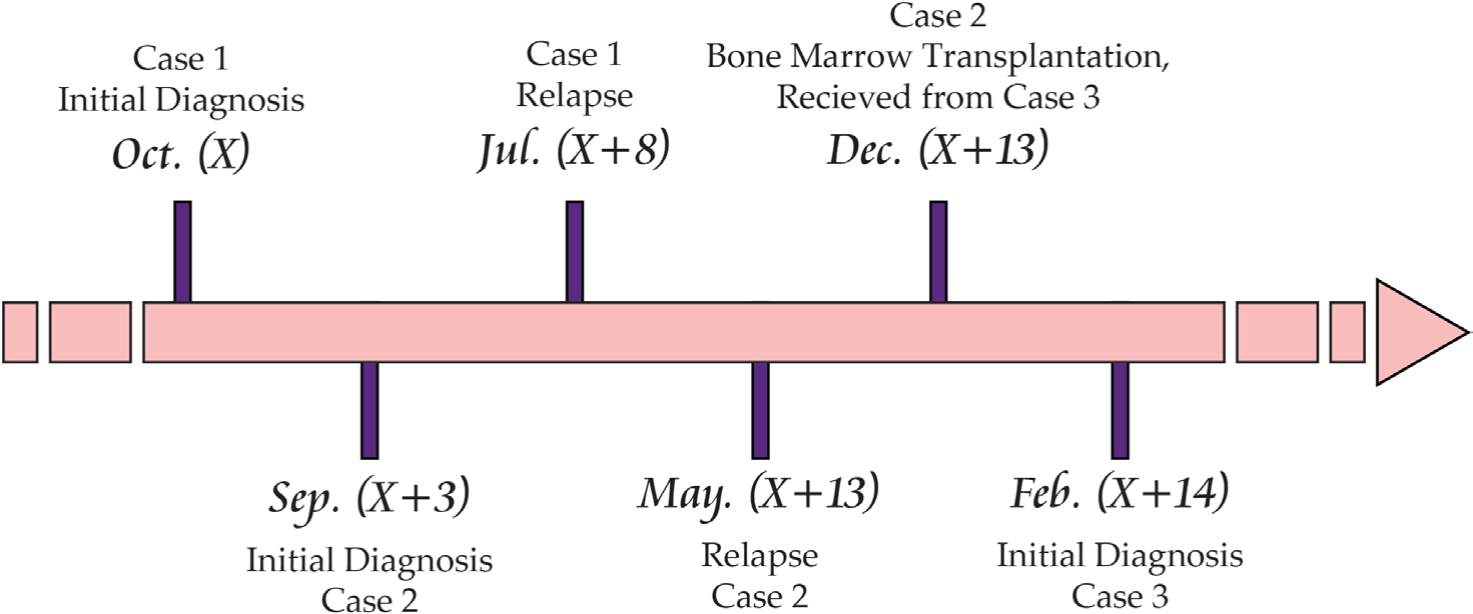
Timeline of clinical events (To ensure patients' anonymity, years were reported relative to the initial diagnosis, where “X” represents the year of Case 1's diagnosis and “+N” denotes N years later).

The youngest child of the family, who had previously provided a hematopoietic stem cell donation to his older brother (Case 2), was admitted when he was 20 years old. The chief complaint was paleness and lethargy. CBC showed normochromic normocytic anemia (Hb = 7.26 g/dL/MCV = 87.2 fL/MCH = 32 pg) and mild thrombocytopenia (Plt = 109 × 10^3^/μL) with 30% blasts in peripheral blood. A hypercellular marrow with 100% cellularity and the presence of sheets of blastic cells positive for CD10, CD19, CD20, CD34, and TdT was reported. The blasts were negative for Sudan‐black stain. Therefore, precursor‐B lymphoblastic leukemia/lymphoma was confirmed. Cytogenetic evaluation displayed a complex karyotype, shown in Figure 1B. This patient was also negative for both p210 and p190 BCR‐ABL/t(9;22)(q34;q11). It should be noted that Cases 2 and 3 were diagnosed as adults with complex karyotypes, so they were considered high‐risk and received hyper‐CVAD, which was both the institutional standard for adults and consistent with risk‐based treatment allocation. Case 3 achieved CR after 2 months of treatment. He is currently on a standard maintenance protocol. Figure 2 represents a timeline of the major clinical events reported in this study. Table 2 compares the demographics and clinical features for easier cross‐reference.

**TABLE 2:** Demographic and clinical features of the reported cases.

Patient ID	Gender (age)	Clinical presentation	Splenomegaly	Treatment protocol	History of relapse	Stem cell transplantation		Current status
						Donate	Recieve	
Case #1	F (10)	Anorexia Weight loss Abdominal pain Fever Nosebleeds Pancytopenia	Yes	AIEOP‐BFM	Yes	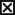	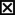	Complete remission
Case #2	M (17)	Epistaxis Pancytopenia	Yes	Hyper‐CVAD	Yes	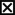	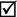	Complete remission
Case #3	M (20)	Paleness and lethargy Anemia Mild thrombocytopenia	No	Hyper‐CVAD	No	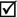	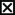	Complete remission

## 4 Conclusion and Results

Fortunately, all three patients have achieved CR and are in a stable condition. This paper emphasizes the need for genetic screening in familial ALL cases, especially those with shared cytogenetic abnormalities. Early detection of at‐risk family members can guide surveillance and preemptive strategies.

## 5 Discussion

Hematological cancers comprise a wide range of neoplastic conditions with various biological and clinical presentations. Some of these cancers show a preference for developing at certain ages. For instance, since fetal factors may contribute to the pathophysiology of the disease, ALL has become the most common malignancy of childhood [10, 11]. ALL usually occurs sporadically and is poorly associated with environmental and genetic risk factors. Nonetheless, prior research has identified ionizing radiation exposure, chemotherapy use, and a history of certain viral infections as environmental risk factors [4]. In 1988, two hypotheses were put forth to implicate infection as a risk factor for ALL [12, 13]. According to these theories, postponed infection or exposure to a particular pathogen may induce an aberrant immune response, which may cause more genetic defects at an age commensurate with elevated lymphoid cell proliferation. However, in our cases, no environmental risk factors were identified, and the past medical history denied severe viral infections and chronic diseases.

Familial acute myeloid leukemia (AML) accounts for approximately 5%–10% of cases, while familial acute lymphoblastic leukemia (ALL) is significantly rarer, representing less than 5% [14, 15]. There is mounting evidence of a substantial genetic susceptibility. Unlike sporadic ALL, familial cases often present with early‐onset disease, higher relapse rates, and shared cytogenetic abnormalities, that is, *ETV6*, *RUNX1*, *KMT2A* rearrangements, and *CDKN2A/B* deletions [16, 17]. Additionally, the familial and inherited character of pediatric B‐ALL has been associated with low penetrance or expression loss of 7p12.2 (IKZF1), 9p12 (CDKN2A/CDKN2B), 10q21.2 (ARID5B), and 14q11.2 (CEBPE) [18]. Due to developments in sequencing technology, several genetically linked abnormalities that contribute to the pathogenesis of pediatric leukemia have been identified, primarily in solitary case reports or family studies. Twin studies and next‐generation sequencing have identified clonal hematopoiesis in predisposed individuals, reinforcing the need for germline testing in families with multiple affected members. Inheritance patterns vary, including autosomal dominant (*PAX5*, *ETV6*), recessive (*BLM*, *ATM*), and polygenic (*ARID5B*, *IKZF1*) mechanisms [19]. Prior research confirms that IKZF1 (7p12.2), PAX5 (9p13), and ETV6 (12p13) are recurrently mutated in familial ALL [20]. Additionally, PAX5 p.Gly183Ser was reported in 3% of familial B‐ALL cases [20]. As mentioned above, mutations in DNA repair genes, tumor suppressor genes, Down syndrome, immune deficiency syndromes, and autoimmune diseases, along with mutations in *CEBPE*, *SH2B3*, *ARID5B*, *IKZF1*, and *CDKN2A/B*, are also listed as the possible risk factors [8].

Monosomy 20, an extremely rare condition in ALL [21, 22], was identified in two of our patients. This finding suggests that, beyond a random secondary event, it may be of pathological relevance in familial cases and needs further investigation in larger familial cohorts. Deletions in 9p (encompassing PAX5 and CDKN2A/B) are also hallmarks of high‐risk B‐ALL [20]. While somatic PAX5 deletions are common, germline PAX5 mutations (e.g., p.Pro80Arg) are rare but clinically significant, as they predispose to familial ALL with early relapse [20]. Hypodiploidy was the other shared cytogenetic abnormality. According to Ghaffari et al., it is associated with a poor overall survival and progression‐free survival [23]. The presence of complex karyotypes with high‐risk markers, such as hypodiploidy, monosomy 7, and *KMT2A* rearrangement in Case 2, further supports the aggressive nature of familial ALL and its association with unfavorable outcomes [16, 24]. These findings align with studies emphasizing the role of germline mutations in PAX5, ETV6, and TP53 in familial ALL [20, 25], reinforcing the need for comprehensive genetic screening in such cases.

The clinical course of the siblings, characterized by relapses in two cases and the development of ALL in the stem cell donor (Case 3), underscores the challenges in managing familial ALL. The full HLA match between Cases 2 and 3 raises questions about the potential transmission of genetic risk factors during stem cell donation, a phenomenon rarely documented in the literature. Regarding donor consideration, the development of ALL in the stem cell donor (Case 3) following transplantation to his HLA‐matched sibling (Case 2) underscores the potential risks associated with using related donors in familial hematological malignancies. These findings are in line with previous literature that noted poorer outcomes for children with familial ALL compared to sporadic cases, with lower 5‐year event‐free survival (EFS) rates, particularly in hypodiploid or KMT2A‐rearranged subtypes [14]. Relapse rates exceed 50%, often driven by chemoresistance and high‐risk genetic profiles (e.g., TP53 mutations) [17].

The presence of three affected siblings may also raise psychosocial and ethical considerations for unaffected family members. These include multifactorial social, emotional, and practical dimensions, such as heightened anxiety about their own cancer risk, fear of social discrimination, financial challenges, challenges in family planning, and uncertainty about genetic predisposition [26, 27, 28, 29]. Therefore, we believe such cases need multidisciplinary support, including appropriate and private psychological and emotional counseling. Furthermore, since asymptomatic family members in high‐risk pedigrees may harbor latent genetic defects, and due to the significant psychosocial and ethical concerns, the American Association for Cancer Research highly recommends pre‐ and post‐testing genetic counseling for individuals with familial predisposition to hematological malignancies. It is also advised to have an initial consultation with a transplant specialist [30].

The familial clustering of ALL in this study mirrors prior reports of inherited syndromes (e.g., Li‐Fraumeni, Bloom syndrome) and germline mutations (e.g., *IKZF1*, *ETV6*) predisposing to leukemia [8]. However, the absence of consanguinity or syndromic features in this family suggests a de novo or polygenic inheritance pattern, which is less well characterized in the literature. The siblings' shared cytogenetic abnormalities provide a potential genetic link, yet the exact mechanism remains elusive. This gap underscores the need for advanced genomic studies, such as whole‐exome sequencing, to identify novel susceptibility loci in familial ALL. In addition, the observation of splenomegaly in our cases highlights the importance of monitoring organ‐related complications, such as spontaneous splenic rupture (SSR) [31]. Future research should assess splenic involvement in familial ALL to determine whether certain genetic subtypes increase SSR risk.

## 6 Limitations

This study described an extremely rare case of familial ALL with no predisposing syndromic defects or previous instances of neoplastic diseases among first‐ or second‐degree relatives. However, it has several limitations: First, cytogenetic data were not available for Case 1, which restricted the ability to compare genetic abnormalities across all siblings. Second, the small sample size limits the generalizability, so the findings should be interpreted cautiously. Third, although high‐throughput sequencing techniques could have provided more detailed genetic insights into germline predisposition in these siblings, resource constraints—both laboratory and financial—as well as the retrospective nature of the study, prevented performing this analysis at the time, and the lack of germline sequencing data impeded identifying specific pathogenic mutations. Future research should incorporate next‐generation sequencing (NGS) to better understand the genetic basis of familial ALL in such cases.

## Author Contributions

**Alireza Khanahmad:** investigation, project administration, writing – original draft. **Mohammad Hossein Khazaee‐Nasirabadi:** investigation, writing – original draft. **Naeem Nikpour:** supervision, writing – review and editing. **Mahmood Khosravi:** conceptualization, writing – original draft.

## Ethics Statement

The research related to human use has been conducted under all the relevant national regulations and institutional policies and in accordance with the tenets of the Helsinki Declaration, and has been approved by the authors' institutional review board or equivalent committee (Ethical Approval Code: IR.KMU.AH.REC.1402.163).

## Consent

Written informed consent was obtained from all patients for the publication of this report.

## Conflicts of Interest

The authors declare no conflicts of interest.

## Data Availability

All data generated or analyzed during this study are included in this published article.
